# Circadian Disruptions in the *Myshkin* Mouse Model of Mania Are Independent of Deficits in Suprachiasmatic Molecular Clock Function

**DOI:** 10.1016/j.biopsych.2017.04.018

**Published:** 2018-12-01

**Authors:** Joseph W.S. Timothy, Natasza Klas, Harshmeena R. Sanghani, Taghreed Al-Mansouri, Alun T.L. Hughes, Greer S. Kirshenbaum, Vincent Brienza, Mino D.C. Belle, Martin R. Ralph, Steven J. Clapcote, Hugh D. Piggins

**Affiliations:** a Faculty of Biology, Medicine, and Health, University of Manchester, Manchester; b School of Biomedical Sciences, University of Leeds, Leeds, United Kingdom; c Lunenfeld-Tanenbaum Research Institute, Mount Sinai Hospital, Toronto, Canada; d Department of Psychology, University of Toronto, Toronto, Canada

**Keywords:** Bipolar, Circadian, Light, Mania, Mood, Suprachiasmatic

## Abstract

**Background:**

Alterations in environmental light and intrinsic circadian function have strong associations with mood disorders. The neural origins underpinning these changes remain unclear, although genetic deficits in the molecular clock regularly render mice with altered mood-associated phenotypes.

**Methods:**

A detailed circadian and light-associated behavioral characterization of the Na^+^/K^+^-ATPase α3 *Myshkin* (*Myk*/+) mouse model of mania was performed. Na^+^/K^+^-ATPase α3 does not reside within the core circadian molecular clockwork, but *Myk*/+ mice exhibit concomitant disruption in circadian rhythms and mood. The neural basis of this phenotype was investigated through molecular and electrophysiological dissection of the master circadian pacemaker, the suprachiasmatic nuclei (SCN). Light input and glutamatergic signaling to the SCN were concomitantly assessed through behavioral assays and calcium imaging.

**Results:**

In vivo assays revealed several circadian abnormalities including lengthened period and instability of behavioral rhythms, and elevated metabolic rate. Grossly aberrant responses to light included accentuated resetting, accelerated re-entrainment, and an absence of locomotor suppression. Bioluminescent recording of circadian clock protein (PERIOD2) output from ex vivo SCN revealed no deficits in *Myk*/+ molecular clock function. Optic nerve crush rescued the circadian period of *Myk*/+ behavior, highlighting that afferent inputs are critical upstream mediators. Electrophysiological and calcium imaging SCN recordings demonstrated changes in the response to glutamatergic stimulation as well as the electrical output indicative of altered retinal input processing.

**Conclusions:**

The *Myshkin* model demonstrates profound circadian and light-responsive behavioral alterations independent of molecular clock disruption. Afferent light signaling drives behavioral changes and raises new mechanistic implications for circadian disruption in affective disorders.

SEE COMMENTARY ON PAGE 775

Bipolar disorder (BPD) is a debilitating mental health condition that affects approximately 0.7% to 0.9% of the population of Western societies (1). BPD is characterized by episodes of depression, euthymia, and mania, but its etiology and neural substrates remain poorly understood (2). Disruption of sleep and circadian rhythms is prevalent in many mental health diseases, including BPD, and as such, underlying circadian systems are implicated within BPD pathophysiology (3). Indeed, treatment of circadian rhythm abnormalities can alleviate symptoms of affective disorders 4, 5, while circadian clock gene polymorphisms represent risk factors across neuropsychiatric conditions (6). Therefore, studying the circadian system may provide insight into the mechanisms and root causes of BPD.

Coordinated circadian rhythms in mammals, including humans, originate from the master circadian clock located in the hypothalamic suprachiasmatic nuclei (SCN) (7). SCN neurons contain an intracellular gene–protein transcription–translation feedback loop (TTFL) that is the molecular basis of circadian timekeeping, and the *Period1/2* (*Per1/2*) genes and their protein products PERIOD1/2 (PER1/2) are key components of this intracellular molecular clock (8). The TTFL drives the SCN neural network to exhibit electrically excited states during the day and relatively quiescent states at night (9). Such variation is key for individual SCN neurons to coordinate their internal clocks, as well as for the SCN to signal and exert temporal control on behavior and physiology (10). Consistent alignment of these central circadian rhythms to the external environmental light/dark (LD) cycle is important for health and well-being (11). Light information signaled directly from intrinsically photosensitive retinal ganglion cells to the SCN is critical in this process (12). This non–image-forming light input pathway, the retinohypothalamic tract, uses the excitatory neurotransmitter glutamate to activate SCN neurons, resetting the phase of the TTFL and ultimately the timing of behavioral and brain states such as sleeping and waking 13, 14. Further, both SCN-dependent and SCN-independent actions of light exert a powerful influence on mood pathology (15).

Intriguingly, in mice, targeted disruption of core TTFL components alters circadian rhythms and consistently elevates the expression of aberrant behaviors resembling those of human affective disorders 16, 17. However, because circadian clock genes are also expressed in mood-regulating brain centers 18, 19, it is challenging to ascribe behavioral deficits directly to specific brain loci (20). Further, in human BPD, it is unclear whether sleep and circadian disruption arise as etiological drivers or as a consequence of wider pathophysiology. To gain insight into these problems, we used the *Myshkin* (*Myk*/+) mouse, which possesses a heterozygous inactivating mutation in the neuron-specific Na^+^/K^+^-ATPase (NKA) α3 subunit, encoded by *Atp1a3*, and models the manic phase of BPD with face, construct, and predictive validity 20, 21. Importantly, this mouse has no known TTFL deficit. We report that *Myk*/+ animals exhibit behavioral circadian rhythm disruption as well as unusually heightened behavioral responses to light and enhanced activation of SCN neurons in vitro to a neurochemical mimic of light input. Intriguingly, we also found that the *Myk*/+ SCN TTFL rhythms are intact, while the electrophysiological output of the *Myk*/+ SCN neural network was damped. Critically, we show that period-lengthening effects on behavior of the *Myshkin* mutation are ameliorated through removal of the light input pathway. Circadian abnormalities in the *Myk*/+ mice arise through alterations in light signaling and processing by the SCN. This model provides new insights into the etiological mechanisms of circadian disruption in animal models of affective disorders that are independent of core circadian clock gene perturbation.

## Methods and Materials

### Animal Housing and Breeding

Adult congenic *Myk*/+ and wild-type (+/+) animals (2–6 months of age) used in this study were bred from pairs (male *Myk*/+ × female +/+) of animals that had been backcrossed on to the C57BL/6NCr strain for 20 generations (20). Pilot investigations revealed no obvious sex differences in behavioral measures in either genotype, so the data from male and female mice were combined (see also Supplemental Figure S1). To generate mice in which the dynamic activities of the molecular clock can be monitored in tissues ex vivo, *Myk*/+ mice were crossed with *mPer2^Luc^* mice bearing a knock-in PER2-luciferase (LUC) construct (referred to here as PER2::LUC mice) (22). Congenic *+/+* × PER2::LUC (*+*/*+*PER2) and *Myk*/+PER2::LUC animals were generated through crosses of heterozygous male *Myk*/+ and female PER2::LUC animals. All behavioral and in vitro studies of mice on the PER2::LUC background were performed on filial 1 generation animals. See Supplemental Methods for further details.

For assessment of daily rhythms in locomotor activity (with or without a running wheel), ingestive behavior (feeding and drinking), and metabolic activity, animals were housed singly as previously described 23, 24. Most studies were conducted under 12-hour LD conditions; however, in some instances animals were assessed under a day-length (16-hour light/8-hour dark cycle) condition or in constant dark (DD) or constant light (LL). These in vivo investigations were conducted using previously established protocols 23, 25; see the Supplement for study-specific details.

For in vitro assessments of electrophysiological activity, calcium transients, and bioluminescence rhythms in PER2::LUC, SCN-containing brain slices were made from adult mice using previously published protocols (26). Whole-cell current-clamp recordings and assessment of calcium transients were performed as previously described 9, 26. Rhythms in whole SCN slice PER2::LUC expression were assessed using luminometry, while single cells in SCN slices were visualized and imaged using a Hamamatsu Image EM9100-13 electron-multiplying-CCD (Hamamatsu, Welwyn Garden City, UK) 27, 28.

### Data Analysis and Statistics

Unless stated otherwise, genotype comparisons were made using two-tailed Student *t* test or by two-way analysis of variance with Sidak post hoc comparisons. For within-genotype comparisons, a one-way analysis of variance was applied with Sidak corrections unless otherwise stated. Nonparametric equivalents and corrections for unequal variances were utilized where appropriate and are detailed in figure captions. The threshold for statistical significance was set at *p <* .05. See the Supplement for further details.

## Results

Circadian mechanisms as well as visual and non–image-forming light pathways influence normal and pathophysiological states including metabolism and mood behaviors (15). As such, we sought to determine if and how murine daily and circadian rhythms are influenced by the *Myshkin* mutation.

**Figure 1:**
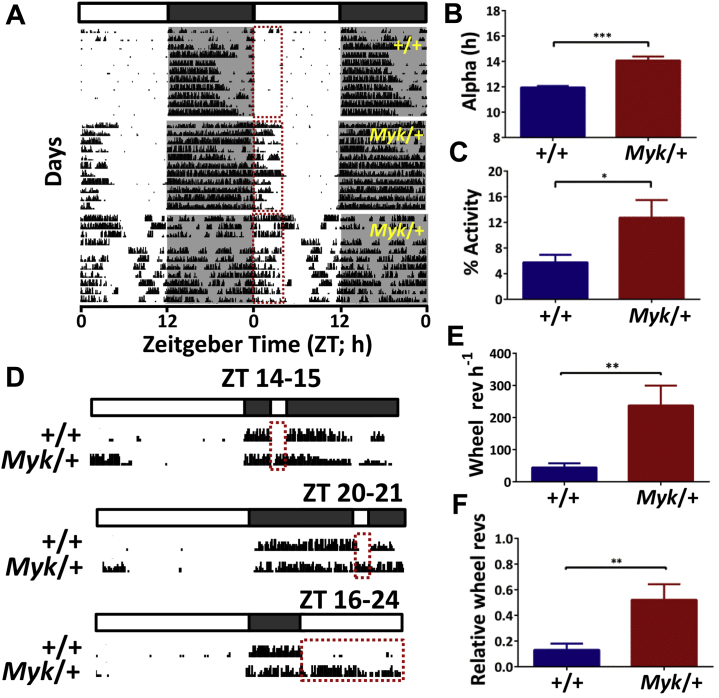
The *Myshkin* mutation alters the suppression of wheel-running behavior by light. **(A)** Example double-plotted actograms from wild-type (*+/+*) (*n =* 37) and *Myk/+* (*n =* 38) mice maintained under 12-hour light/dark conditions. Red boxes indicate typical region when “tails” in *Myk/+* light-phase activity occur. Gray-shaded areas of actograms delineate lights off (dark). Zeitgeber time 0 (ZT0) = lights on; ZT12 = lights off. **(B)** Alpha duration under these 12-hour light/dark conditions is elongated in *Myk/+* animals (*+/+*: 11.95 ± 0.12 hours, *Myk/+*: 14.07 ± 0.32 hours; *p <* .0001). **(C)** Percentage of total daily activity in the lights-on phase is increased by the *Myshkin* mutation (*+/+*: 5.8 ± 1.2%, *Myk/+*: 12.7 ± 2.7%; *p =* .043). **(D)** Example single-plotted actograms showing the presence (*+/+* mice) and absence (*Myk/+* animals) of negative masking responses to 1-hour or 8-hour light pulses given during the lights-off phase. **(E)** Wheel revolutions per hour exhibited during these 1-hour or 8-hour light pulses are increased by the *Myshkin* mutation (*+/+*: 45 ± 13 revolutions/hour^−1^ (rev h^−1^), *Myk/+*: 239 ± 62 rev h^−1^; *p =* .005). **(F)** Locomotor activity during light pulse (running wheel revolutions) normalized to each animal’s daily mean is higher in *Myk/+* mice (*+/+*: 0.13 ± 0.05 relative wheel revolutions, *Myk/+*: 0.52 ± 0.12 relative wheel revolutions; *p* = .002). Data are plotted as mean ± SEM. *p < .05; ** *p <* .01; ***p < .001.

When singly housed under standard 12-hour LD conditions, the mania phenotype of *Myk*/+ mice was characterized by an inability to restrict the normal nocturnally elevated wheel-running activity to the dark phase. Instead, *Myk*/+ animals sustained vigorous activity into the first 3 to 4 hours of the lights-on phase (Figure 1A). Consequently, in comparison with *+/+* animals, the duration of the daily active (alpha) phase was significantly lengthened by ∼2.1 hours (Figure 1B), with the percentage of wheel running occurring during the day elevated by ∼6.9% (Figure 1C) in *Myk*/+ mice. Some *Myk*/+ animals also showed unstable LD rhythms, with activity onsets variably preceding the initiation of the dark phase (Figure 1A). The effects did not rely on the provision of a running wheel, because monitoring of general locomotor activity via infrared detector indicated near-identical disruption in *Myk*/+ animals (Supplemental Figure S4A–D). Similar effects of the *Myshkin* mutation on wheel-running activity were observed in a separate cohort of female mice (Supplemental Figure S1A–E).

In humans, extending daily exposure to light (>14 hours per 24 hours) associated with summer months can exacerbate the symptoms of mania 29, 30. When transferred into longer day length (16 hours light/8 hours dark), *Myk*/+ mice exhibited longer alpha, weaker rhythms, and ∼40% higher wheel running in the light phase than *+/+* mice (Supplemental Figure S2A–D). This reveals that the *Myshkin* mutation compromises behavioral consolidation and that increasing the duration of the lights-on phase overtly disrupts rhythmic control of behavior.

**Figure 2:**
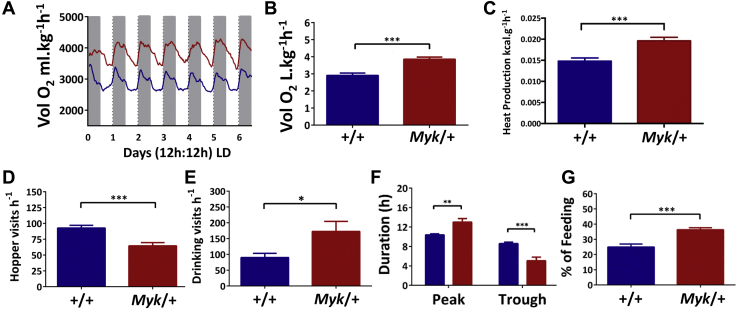
Altered metabolic rhythms in *Myk/+* mice. **(A)** Smoothed traces of wild-type (*+/+*) mice (blue; *n =* 10) and *Myk/+* mice (red; *n =* 12) maximal oxygen consumption (Vol O_2_) over 6.5 days. Gray-shaded columns delineate lights off (dark). **(B)** Mean Vol O_2_ consumption is elevated in *Myk/+* animals (*+/+*: 2.91 ± 0.14 L/kg/hour, *Myk/+*: 3.86 ± 0.13 L/kg/hour; *p <* .0001). **(C)** Mean hourly heat production is elevated in the *Myk/+* mice (+/+: 0.0148 ± 0.0007 kcal/g/hour, *Myk/+*: 0.196 ± 0.0008 kcal/g/hour; *p =* .0003). **(D)** Altered ingestive behaviors in *Myk/+* animals. Mean hourly food hopper visits were reduced by the *Myshkin* mutation (*+/+*: 92.6 ± 4.3 visits, *Myk/+*: 64.6 ± 5.1 visits; *p =* .0005). **(E)** Mean hourly visits to drinking spout were increased by the *Myshkin* mutation (*+/+*: 90.0 ± 13.2 visits/hour, *Myk/+*: 172.6 ± 31.7 visits/hour; *p =* .03). **(F)** Altered duration of daily peak (*+/+*: 10.37 ± 0.23 hours, *Myk/+*: 13 ± 0.75 hours; *p =* .003) and nadir (*+/+*: 8.59 ± 0.30 hours, *Myk/+*: 5.05 ± 0.75 hours; *p =* .0004) in Vol O_2_ activity. **(G)** The *Myshkin* mutation increases percentage of daily feeding occurring during the lights-on phase (*+/+*: 25.0 ± 1.9%, *Myk/+*: 36.3 ± 1.3%; *p <* .0001). Data in panels **(C**–**G)** are plotted as mean ± SEM. *p < .05, **p < .01, ***p < .001. LD, light/dark.

Circadian disruption and mental illness can alter body weight regulation 31, 32, 33. Indeed, bipolar patients with mania can exhibit elevated basal metabolic rate (34), so we subsequently profiled metabolic activity in *Myk*/+ and *+/+* mice. Using indirect calorimetry and monitoring of ingestion activity for 6.5 days under a 12-hour LD cycle, *Myk*/+ animals were found to exhibit elevated basal metabolic rate, heat production, and drinking activity (Figure 2A–C, E). The duration of their elevated metabolic activity was sustained into the lights-on phase (Figure 2A, F); ingestive behavior was increased during the lights-on phase but was reduced over 24 hours (Figure 2D, G).

In nocturnal rodents, light exposure typically suppresses locomotor behaviors (negative masking), but when exposed to 1-hour or 8-hour pulses of light during the night, *Myk*/+ mice maintained activity or increased wheel-running behavior during the pulses, while *+/+* animals reduced locomotor activity (Figure 1D–F). Further, when released into an illuminated (∼1.5 μW/cm^2^) open-field test arena for 15 minutes during the early night (Zeitgeber time 15–18 [ZT15–18]), hyperlocomotor activity (as assessed by distance traversed) was marked in *Myk*/+ but not in +/+ mice (Supplemental Figure S3). Therefore, unlike other nocturnal rodents such as the Syrian hamster (35) as well as mice with TTFL mutations (36), *Myk*/+ mice do not exhibit pronounced negative masking, an effect overtly manifested in wheel running during the day.

**Figure 3:**
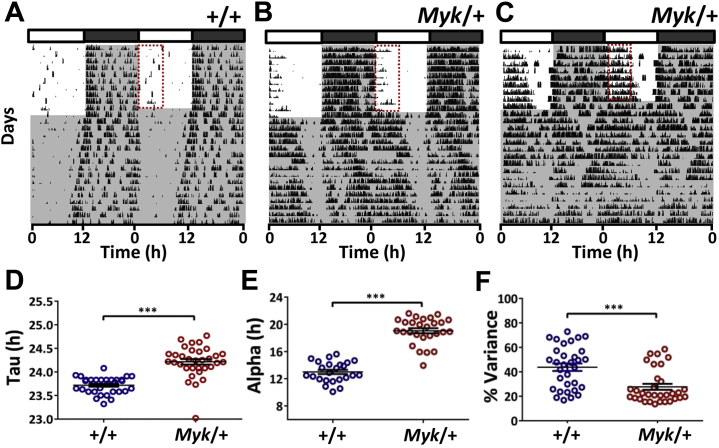
Altered free-running rhythms in *Myk/+* mice. **(A–C)** Example double-plotted actograms of wild-type (*+/+*) and *Myk/+* animals released into constant dark (indicated by gray shading) following entrainment to 12-hour light/dark cycle. Most *Myk/+* mice maintained rhythms in constant dark **(B)**, but exceptionally, some **(C)** became arrhythmic. Red rectangles delineate timing of typical elevated wheel running in the *Myk/+* mice. **(D)** The *Myshkin* mutation lengthened free-running period (*+/+*: 23.72 ± 0.03 hours, *Myk/+*: 24.22 ± 0.02 hours; *p <* .0001) and **(E)** alpha duration (*+/+*: 13.14 ± 0.26 hours, *Myk/+*: 18.99 ± 0.37 hours; *p <* .0001). **(F)** The amplitude of daily wheel-running rhythms is reduced by the *Myshkin* mutation (as measured by chi-square periodogram; *+/+*: 43.8 ± 3.1% variance, *Myk/+*: 27.7 ± 2.4% variance; *p =* .001). Data in panels **(D–F)** are graphed as scatter plots with mean ± SEM. ***p < .001.

Because *Myk*/+ mice exhibited disrupted rhythms under LD conditions, their intrinsic circadian rhythms in wheel-running behavior were initially assessed over 14 days in the absence of light (DD). Consistent with a previous report (20), behavioral rhythms of *Myk*/+ mice differed significantly from those of +/+ animals. *Myk*/+ mice displayed a lengthened period (Figure 3A–D; ∼24.2 hours vs. ∼23.5 hours) and an unusually elongated active phase (Figure 3E: alpha; ∼19.0 hours vs. ∼13.1 hours), while the amplitude, or strength (percent variance as measured by chi-square periodogram), of their behavioral rhythms was also markedly reduced (Figure 3F). In a separate all-female cohort, *Myk*/+ animals also showed similar changes in circadian rhythms of wheel running in DD (Supplemental Figure S1A, B, F–H). These effects of the *Myshkin* mutation were not dependent on the provision of a running wheel 37, 38, as similar changes in general locomotor activity rhythms were exhibited by animals monitored with a passive infrared system without a functioning running wheel (Supplemental Figure S4A, B, E, F).

When assessed for an additional 14 to 21 days in DD, all *+/+* animals sustained rhythmic wheel-running activity, whereas that of some *Myk*/+ mice (*n =* 3 of 38; 8%) weakened and they became circadianly arrhythmic (Figure 3C). Additionally, some *Myk*/+ animals (but no *+/+* mice) exhibited gradual unusual changes in free-running period (*n =* 8 of 38; 21%) either spontaneously or following transfer to a clean cage (Supplemental Figure S5A–C). Because rhythm amplitude and free-running stability are metrics for the output strength of the circadian system, this indicates that *Myk*/+ animals possess diminished and unstable central circadian regulation of behavior and physiology.

**Figure 4:**
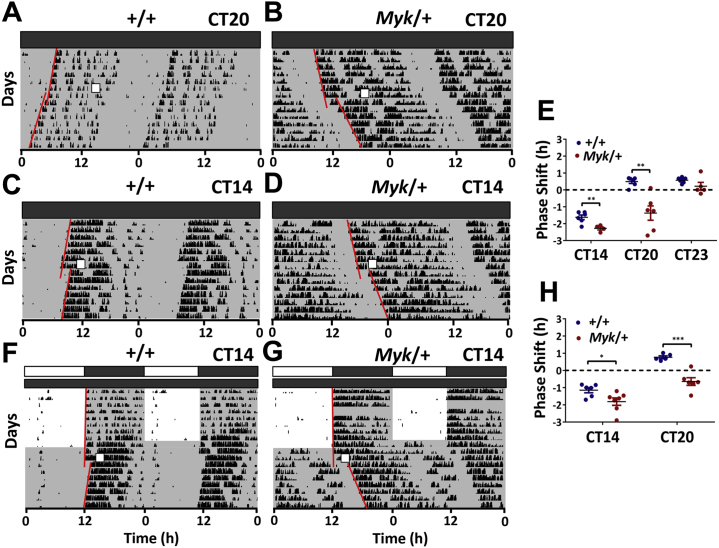
Enhanced circadian resetting responses to light in *Myk/+* mice. **(A, B)** Example double-plotted actograms showing resetting effects of a 1-hour light pulse (Aschoff type I protocol) given under constant dark conditions to wild-type (*+/+*) and *Myk/+* animals (white boxes indicate light pulse) during the late subjective night (circadian time [CT] 20). Note the opposing direction (delay) of the phase shift in *Myk*/+ activity onset compared with the advance in activity onset of the +/+ animal. **(C, D)** Example double-plotted actograms illustrating the shifting effects of a 1-hour light pulse (Aschoff type I protocol) given early in the subjective night (CT14) on the free-running rhythms of *+/+* and *Myk/+* animals. *Myk/+* animals exhibited larger phase delays at CT14. **(E)** Individual responses to 1-hour light pulses given under Aschoff type I protocol (CT14, *+/+*: −1.6 ± 0.1 hours, *Myk/+*: −2.3 ± 0.1 hours, *p =* .008; CT20, +/+: 0.5 ± 0.1 hours, *Myk/+*: −1.3 ± 0.4 hours, *p =* .002; CT23, +/+: 0.6 ± 0.1 hours, *Myk/+*: 0.2 ± 0.2 hours, *p =* .14). **(F, G)** Double-plotted actograms showing the phase resetting responses of +/+ and *Myk/+* mice to 1-hour light pulse given early in the subjective night (CT14) following transfer from light/dark to constant dark conditions (Aschoff type II protocol). Animals were exposed to a 1-hour light pulse within 48 hours following release into constant dark. **(H)** Individual responses to 1-hour light pulses presented under Aschoff type II protocol (CT14, *+/+*: −1.1 ± 0.1 hours, *Myk*/+: −1.8 ± 0.2 hours, *p =* .03; CT20, *+/+*: 0.76 ± 0.08 hours, *Myk*/+: −0.48 ± 0.18 hours, *p =* .0001). Panels **(E)** and **(H)** are graphed as scatter plots with mean ± SEM. *p < .05, **p < .01, ***p < .001.

For rodents in DD, light exposure during the night shifts subsequent onsets of activity; early night exposure (circadian time 14–18 [CT14–18]) delays circadian rhythms, while light given later in the night (CT20–24) advances rhythm onsets (39), so we next sought to determine if the *Myshkin* mutation affected photic resetting of the circadian system. Mice were released from 12-hour LD conditions into DD and, after 14 days, were exposed to a 1-hour light pulse either late (CT20–21; Figure 4A, B) or early (CT14–15; Figure 4C, D) in their active subjective night (an Aschoff type I protocol). The subsequent phase of the onset of their wheel-running rhythms was then measured (23). When exposed to light pulses at CT14, *Myk*/+ mice showed much larger phase delays than +/+ mice (∼−2.32 hours vs. ∼−1.6 hours, *p =* .008; Figure 4C–E). Light pulse treatment at CT20 evoked phase delays in *Myk*/+ activity that were unusual, as they were of the opposite direction to typical advances elicited at this time in +/+ mice (∼−1.3 hours vs. +0.5 hours, *p* ≤ .02; Figure 4A, B, E). No genotype differences were found with a light pulse given near the subjective night-day transition (CT23; Figure 4E). This experiment was repeated with 1-hour light pulses given within 48 hours following release from 12-hour LD into DD (an Aschoff type II protocol) with similarly altered resetting patterns observed (Figure 4F–H). This indicates that the altered phase shifts to light observed in *Myk*/+ mice do not emerge as a consequence of long-term adaptation to DD. Thus, the *Myshkin* mutation both accentuates and alters the temporal pattern of the resetting effects of light on murine rhythms in behavior.

**Figure 5:**
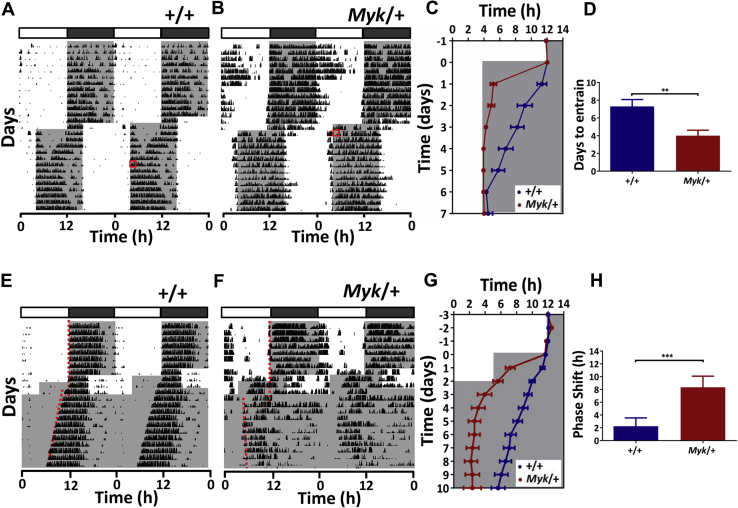
Rapid adaptation to simulated jetlag in *Myk/+* mice. **(A, B)** Double-plotted actograms of mice exposed to simulated jetlag protocol; here the light/dark (LD) cycle was advanced by 8 hours. Red boxes indicate the time point at which the animals were designated as having re-entrained to the new LD cycle. Gray-shaded areas in actograms and in panels **(C)** and **(G)** delineate lights off (dark). **(C)***Myk*/+ mice readjust their daily onset in wheel-running activity to the new LD phase more rapidly than do wild-type (+/+) mice. Note the large readjustment in the onset of daily wheel running observed in the *Myk*/+ mice on day 1 following the advance in the LD cycle. **(D)***Myk*/+ mice take fewer days to re-entrain their wheel-running activity to the new LD cycle than do +/+ animals (+/+: 6.5 ± 0.7 days vs. *Myk*/+: 3.6 ± 0.6 days; *p =* .006). **(E, F)** Double-plotted example actograms of +/+ and *Myk*/+ animals responding to transient jetlag. To validate resetting of the circadian system to simulated jetlag seen in panels **(A, B)**, mice were exposed to a 7-hour advance of the 12-hour LD cycle for 48 hours and then released into constant dark. *Myk*/+ animals show a large magnitude advance in the timing of their wheel-running activity. **(G)** The larger readjustment of the onset in daily wheel running of *Myk*/+ mice is sustained in constant dark, indicating that the circadian system of these animals has reset to a much larger extent than +/+ mice. **(H)** Mean phase shift on first 24 hours in constant dark is significantly larger in *Myk*/+ mice (+/+: 2.2 ± 0.5 hours, *Myk/+*: 8.3 ± 0.8 hours; *p <* .0001). Data in panels **(C, D, G, H)** plotted as mean ± SEM. **p < .01, ***p < .001.

We next assessed if the *Myshkin* mutation influenced how mice respond to simulated jetlag. Jetlag and other external disruptors to normal activity rhythms are associated with the presentation of episodes in BPD, and therefore sensitivity to external perturbation represents an important measure 40, 41. In response to the 8-hour advance (Figure 5A–D) or delay (Supplemental Figure S5D, E) of the LD cycle, *Myk*/+ mice rapidly altered their daily pattern of wheel running within 2 to 4 days, whereas +/+ animals took 6 to 8 days to resynchronize. Comparable rapid resynchronization to an 8-hour advance in the LD cycle was also observed in a separate cohort of female *Myk*/+ mice (Supplemental Figure S1A, B, I, J). To directly test if the *Myshkin* mutation enhances the photic resetting capabilities of the neural circadian system, animals were next subjected to a transient 7-hour advance of the LD cycle for 2 days then released into free-running DD conditions (Figure 5E, F). Again, *Myk*/+ mice demonstrated unusually large advances in the new phase of their activity onsets, and on the first day in DD these were of much greater magnitude (∼8.3 hours vs. ∼2.2 hours) than were those shown by +/+ animals (Figure 5G, H). This finding indicates that mechanisms that normally brake the circadian system to prevent extraordinarily large resetting are dysfunctional in *Myk*/+ animals 42, 43.

In rodents, exposure to LL suppresses wheel-running behavior and lengthens the period of circadian rhythms 24, 44, so we next assessed how *Myk*/+ mice adapt to LL. Consistent with previous research, all 8 +/+ animals showed a suppression of wheel running in LL (reduced by ∼90% from LD) and exhibited free-running rhythms with a period of ∼25 hours (Supplemental Figure S1A, B, K). *Myk*/+ animals also showed longer period rhythms in LL (∼25 hours), but most (5 of 7) exhibited markedly elevated wheel running in LL (∼403% increase from LD). Therefore, while the period-lengthening effects of LL are observed in *Myk*/+ animals, some individuals sustain increased rather than decreased locomotor activity.

**Figure 6:**
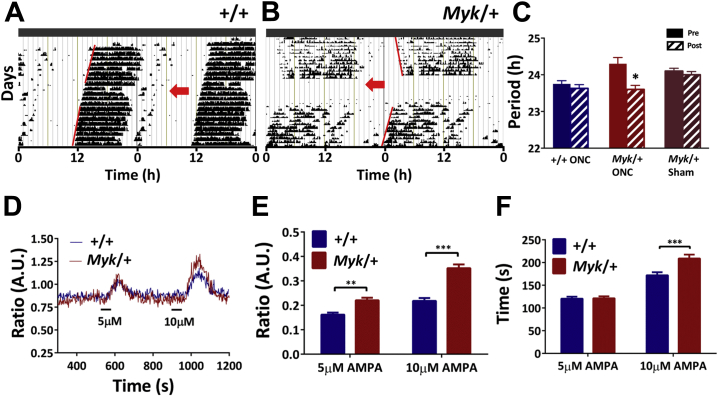
Altered retinal signals and excitatory processing within the suprachiasmatic nuclei (SCN) underlie *Myk/+* circadian behavioral phenotypes. **(A, B)** Double-plotted wheel-running actograms illustrating the effects of optic nerve crush (ONC) in wild-type (*+/+*) and *Myk/+* animals maintained in constant dark conditions. Red arrows delineate the day of ONC. Red lines are fitted through activity onsets pre- and postsurgical intervention. The free-running period of *Myk/+* mice was restored by ONC but not sham procedure. **(C)** Mean period of cohorts undergoing ONC or sham procedure (*+/+* ONC *n =* 6: ∼−0.1 hours, *p =* .13; *Myk/+* ONC *n =* 6: −0.75 hours, *p =* .025; *Myk/+* sham *n =* 4: ∼−0.1 hours, *p =* .12). **(D)** Example responses of single SCN neurons loaded with Fura-2 to alpha-amino-3-hydroxy-5-methyl-4-isoxazole propionic acid (AMPA) (5 and 10 μM) tested during subjective night (*+/+*: *n =* 88, *Myk/+*: *n =* 148). **(E)** When tested during the subjective night (Zeitgeber time 14–18 [ZT14–18]), *Myk/+* SCN neurons exhibited larger AMPA-evoked cellular increases in intracellular Ca^2+^ than did *+/+* SCN neurons (two-way analysis of variance: interaction *p <* .0001; *+/+* 5 μM: 0.16 ± 0.01 arbitrary units (A.U.), *Myk/+* 5 μM: 0.22 ± 0.01 A.U., Sidak *p =* .0029; *+/+* 10 μM: 0.22 ± 0.01 A.U., *Myk/+* 10 μM: 0.35 ± 0.01, *p <* .0001). **(F)** During subjective night (ZT14–18), the washout duration following AMPA treatment (10 μM) was longer in the *Myk/+* neurons compared with *+/+* SCN neurons (*+/+* 5 μM: 121 ± 4 seconds, *Myk/+* 5 μM: 121 ± 4 seconds, *p =* .99; *+/+* 10 μM: 172 ± 4 seconds, *Myk/+* 10 μM: 209 ± 8 seconds, *p =* .0007). Data in panels **(C**, **E**, **F)** are plotted as mean ± SEM. *p < .05, **p < .01, ***p < .001.

The NKA α3 pump is localized to several central nervous system structures, including retinal ganglion cells whose axons project along the optic nerve 45, 46, so we next investigated if retinal input to the SCN contributed to the altered circadian rhythms of *Myk*/+ mice. To do so, mice free running in DD either underwent a sham surgical procedure or had their optic nerves crushed (see the Supplement for procedural details). Optic nerve crush in *Myk*/+ mice markedly shortened their circadian period by ∼−0.7 hours to ∼23.6 hours, while *+/+* animals showed no obvious change in circadian period (Figure 6A–C). Similarly, *Myk*/+ mice undergoing sham surgical procedure showed no change in free-running period (Figure 6C). This indicates that aberrant intrinsic activity of the light input pathway to the SCN contributes to the period-lengthening effect of the *Myshkin* mutation.

Glutamate is the main neurochemical of the retinal input pathway to the SCN, and stimulation of the alpha-amino-3-hydroxy-5-methyl-4-isoxazole propionic acid (AMPA)–type glutamate receptors excites SCN neurons, elevates intracellular Ca^2+^, and can shift the phase of behavioral rhythms 47, 48. To determine if acute responses of SCN neurons to glutamate were altered by the *Myshkin* mutation, the actions of AMPA (5–20 μM) on intracellular Ca^2+^ were assessed in *+/+* and *Myk*/+ hypothalamic SCN brain slices. Neurons were loaded with the fluorescent calcium indicator dye Fura-2-acetoxymethyl ester to enable recording of somatic intracellular Ca^2+^ changes across populations of single SCN cells (Supplemental Figure S6A).

During the day (ZT4–10), AMPA treatments evoked changes in intracellular Ca^2+^ of similar magnitude and duration in both genotypes (Supplemental Figure S6B, C). However, with applications made during the night (ZT14–18), AMPA elicited increases in intracellular Ca^2+^ that were significantly larger in *Myk*/+ neurons compared with +/+ SCN neurons (Figure 6D, E). At this time, response magnitude was dose dependent (5 and 10 μM), with baseline recovery from the 10-μM application taking significantly longer in the *Myk*/+ SCN neurons (Figure 6F). Such changes in AMPA responses indicate that, in addition to altering retinohypothalamic tract activity, the *Myshkin* mutation enhances the processing of a neurochemical mimic of this light input pathway to the SCN.

**Figure 7:**
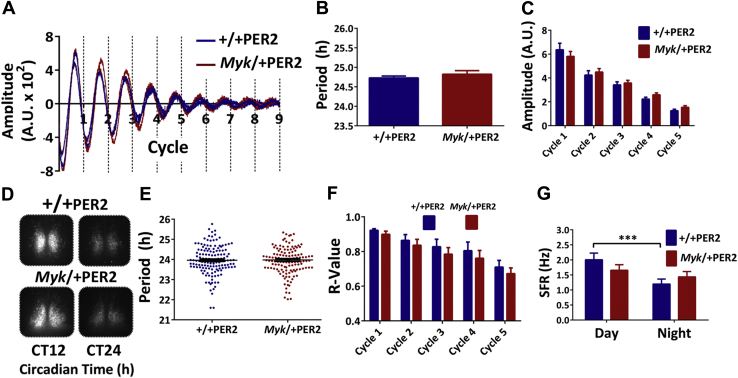
The *Myshkin* mutation does not affect bioluminescent rhythms of PER2::LUC in whole suprachiasmatic nuclei (SCN) brain slices or single cells, but rather damps electrophysiological activity. **(A)** Example luminometric recordings of rhythms of PER2::LUC output of whole SCN tissue explants from *+/+*PER2::LUC (+/+PER2) and *Myk/+*PER2::LUC (*Myk/+*PER2). **(B)** No genotype differences were detected in the period of PER2::LUC oscillations (*+/+*PER2: 24.73 ± 0.05 hours, *Myk/+*PER2: 24.82 ± 0.09 hours; *p =* .39). **(C)** Peak amplitude of PER2::LUC rhythms across the first 5 days (24-hour cycles) in culture did not differ between the genotypes (two-way analysis of variance: genotype *p =* .78; interaction *p =* .13). **(D)** Example of single-cell imaging from *+/+*PER2 (*n =* 4) and *Myk/+*PER2 (*n =* 4) explants at two time points over initial 24 hours ex vivo. **(E)** The period of single-cell rhythms did not differ between the genotypes (*+/+*PER2: *n =* 140, 23.96 ± 0.06 hours; *Myk/+*PER2: *n =* 132, 23.96 ± 0.07 hours; *p =* .99). **(F)** The synchrony (*R*) between single cells in the SCN slices did not differ between the genotypes at any of the 5 days (5 × 24-hour cycles) ex vivo (two-way analysis of variance: genotype *p =* .70). **(G)** Mean spontaneous firing rate (SFR) from whole-cell current-clamp recordings made over a 24-hour cycle. Day/night variation in SFR is seen across *+/+* SCN neurons but not *Myk/+* SCN neurons (two-way analysis of variance: time *p =* .0079; *+/+* day: 2.0 ± 0.2 Hz, *+/+* night: 1.2 ± 0.2 Hz, *p =* .0009; *Myk/+* day: 1.6 ± 0.2 Hz, *Myk/+* night: 1.4 ± 0.2 Hz; *p =* .31). Data in panel **(E)** graphed as scatter plot with mean ± SEM. Data in panels **(B**, **C**, **F**, **G)** plotted as mean ± SEM. ***p < .001. A.U., arbitrary units; CT, circadian time.

To gain further insight into the etiology of circadian disturbances arising from the *Myshkin* mutation, we next investigated the anatomy and TTFL molecular pacemaking of the *Myk*/+ SCN. The SCN exhibit characteristic, spatially distinct neuropeptide topography. Immunohistochemical staining for major SCN neuropeptides, vasoactive intestinal polypeptide and arginine vasopressin, showed no overt genotype differences in the pattern of expression (Supplemental Figure S7). Molecular clock activity was then tracked through expression of the circadian clock protein PER2 via PER2::LUC expression in SCN explants (Supplemental Figure S8). The characteristics of circadian rhythms in wheel running seen in *Myk*/+ mice (increased circadian period and alpha) were maintained in *Myk*/+PER2::LUC mice (Supplemental Figure S8A–E). Surprisingly, in SCN explants cultured ex vivo, no overt genotype differences were found in period or amplitude of whole-tissue PER2::LUC expression (Figure 7A–C)*.* Similarly, in SCN explants imaged at single-cell resolution, neither the period nor the damping rate of single-cell rhythms nor the intercellular synchrony within SCN slices differed between the genotypes (Figure 7D–F). The persistence of +/+PER2*-*like rhythms ex vivo indicates that, in isolation, the *Myk*/+ SCN is a stable pacemaker. Together with the findings from the optic nerve crush experiment, this reinforces the idea that, in vivo, afferent signals such as those coming from the eye act to diminish the *Myk*/+ SCN’s control of physiology and behavior.

Neurons of the SCN control behavior and physiology in part by varying their spontaneous firing rate (SFR), with higher frequency discharge during the day than at night (49). Because neurophysiological studies suggest that the NKA pump influences SCN neurons (50), and because NKA α3 subunits affect membrane excitability 51, 52, we next made whole-cell electrophysiological recordings and assessed the electrical activity of *+/+* and *Myk*/+ SCN neurons maintained in brain slices. SCN neurons exhibit distinct electrophysiological states 9, 53 and these were evident in both *+/+* and *Myk*/+ SCN recordings, but no genotype differences were detected in most passive properties of these neurons (Supplemental Table S1). However, comparison of the SFR of *Myk*/+ and +/+ SCN neurons based on the time of recording indicated clear genotype-related differences. Unexpectedly, the mean SFR of *Myk*/+ SCN neurons did not differ from day to night recordings, whereas +/+ SCN neurons had significantly higher SFR during the day as compared with the night (Figure 7G). Day/night variation in SFR is a key characteristic of the SCN network both in vitro and in vivo, and because the TTFL appears to be intact in the *Myk*/+ SCN, this damping in *Myk*/+ SCN neuronal activity most likely arises from exposure to altered photic afferent signals.

## Discussion

Here we have demonstrated that, in addition to increases in the period and active phase duration of circadian rhythms in behavior, the *Myk*/+ mouse exhibits instability in behavioral rhythms and unusually heightened circadian resetting/re-entrainment responses to light. Other mouse models expressing mania-like states, including *Clock*^*Δ19*^*, Reverbα*^*–/–*^, and DAT^–/–^ mice, also show heightened phase-shifting responses to light 54, 55, 56. Interestingly, in patients with mania, locomotor rhythms may also weaken with increasing severity of manic symptoms (57), and sensitivity to both white and blue light is heightened during manic episodes 58, 59, 60. Similar to bipolar patients 61, 62, *Myk*/+ mice exhibit both altered sleep patterns (20) and circadian rhythm disturbance as well as elevated metabolic activity (34). Notably, the nocturnally elevated metabolic rate and locomotor activity of *Myk*/+ mice were not suppressed by light, indicating the absence of negative masking. Consistent with this, *Myk*/+ mice did not behaviorally adjust to a long day length, and in LL they increased wheel running, whereas +/+ mice markedly reduced it. Indeed, even in the absence of light, alterations in retinal afferents were sufficient to drive circadian behavioral disruption, because removal of afferent photic input to the SCN in vivo restored the circadian period of behavioral rhythms. Further, when cultured in vitro and assessed in isolation from retinal inputs, *Myk*/+ single-cell PER2 rhythms and whole-SCN explants behaved as stable pacemakers, comparable to +/+ SCN tissue. This indicates that the SCN molecular clock is largely intact in this mouse model of mania, a finding concordant with the observation that the molecular clock is also intact in fibroblasts from patients with BPD (63). However, *Myk*/+ SCN slices lacked day/night variation in neuronal firing rate. Importantly, the low amplitude, long period behavioral rhythms, and damped SCN electrical activity of *Myk*/+ mice resemble similar measures made from +/+ mice exposed to long day lengths or LL 44, 64, 65, 66. These findings reveal an important role for light inputs to the SCN in this mouse model of mania.

NKA α3 is localized to central and peripheral neurons, including retinal ganglion cells 46, 67, and glutamate is the main neurotransmitter of image- and non–image-forming pathways from the eye 12, 49. Both SCN and extra-SCN sites are implicated in masking effects of light 68, 69 and the absence of negative masking in *Myk*/+ mice presumably arises as a consequence of altered glutamatergic signaling at these sites. Glutamatergic synapses are linked with mood disorders (70), and it is suggested that measures of glutamate in the brain vary in BPD, becoming elevated during mania and reduced in episodes of depression (71). Further, mood stabilizers used to treat BPD, such as lithium and valproate, act to restore glutamate levels 72, 73 and can reduce sensitivity to light and directly alter SCN function 74, 75. Interestingly, colocalization and functional coupling between NKA α3 and glutamate transporters has been demonstrated in rat brain, with α3 having similar neuronal localization to excitatory amino acid transporter 2/glutamate transporter 1, the most abundant subtype of glutamate transporter in the central nervous system (76). Previously, Kirshenbaum *et al.* (20) found that the duration of glutamate-evoked [Ca^2+^]_i_ transients was prolonged in cultured cortical neurons from *Myk*/+ mice (20), raising the possibility that this mutation alters glutamatergic signaling in other brain pathways. Because metabolic rate is elevated in *Myk*/+ mice, it is plausible that the *Myshkin* mutation influences energy balance centers in the mediobasal hypothalamus (77).

In high-firing hippocampal and cerebellar neurons, loss or reduction of NKA α3 is associated with neuronal hyperexcitablity 20, 51, 78, 79. *Atp1a3* is expressed in the SCN (80), but *Myk*/+ SCN neurons that have a reduction in functional NKA α3 show damped daytime firing rate. To discharge action potentials, high-firing cells can require considerable adenosine triphosphate, and a likely consequence of a reduction in NKA pump activity is ionic imbalance and chronic depolarization (81). Because individual SCN neurons are comparatively low firing (typically <5 Hz), this suggests that their adenosine triphosphate requirements are low, such that a reduction in NKA α3 activity does not drive the cell into a chronic depolarized state. Indeed, because some classes of intrinsically photosensitive retinal ganglion cells can spontaneously fire at high frequencies (70–90 Hz) (82), it is probable that they are more readily hyperexcited through the reduction in NKA α3 activity, as is evidenced by the associated changes in retinal input to the SCN in *Myk*/+ animals.

NKA α subunits (α1–α3) are associated with BPD, although subunit-specific roles in pathology remain to be defined 20, 83, 84. A heterozygous missense mutation (V129M) in NKA α3 was identified in a 9-year-old boy with DSM-5 schizophrenia and a clinical presentation including mood swings (85). Multiple *Atp1a3*-specific mutant mouse models exhibit variable presentations of neurological deficits 86, 87, but one key commonality is that all show heightened psychomotor states. Missense mutations in NKA α3 have been identified in other neurological conditions including rapid-onset dystonia parkinsonism, alternating hemiplegia of childhood (AHC), and CAPOS (cerebellar ataxia, areflexia, pes cavus, optic atrophy, and sensorineural hearing loss) syndrome 88, 89. Patients with rapid-onset dystonia parkinsonism show greater incidence of mood and psychotic symptoms than control family members (90). AHC patients are easily aroused and prone to behavioral and psychiatric symptoms, such as impulsivity, lack of attention control, and episodes of hyperactivity (H. Rosewich, M.D., personal communication, July 6, 2016). Clinical presentation of AHC is sensitive to changes in lighting and sleep induction. Indeed, acute presentation of AHC symptoms such as hemiplegic attacks can be triggered by stimulation of the optic system (H. Rosewich, personal communication) and attenuated through occlusion of the eyes (91). In addition to circadian-associated behaviors, therefore, our findings support a wider role of light and retinal signaling in the pathology of *ATP1A3*-related disorders. As the activity of NKA α3 was recently found to be impaired by its aberrant association with misfolded and aggregated amyloid-β in Alzheimer’s disease (92) and α-synuclein in Parkinson’s disease (93), NKA α3 dysfunction may also contribute to the circadian sleep disturbances in these common age-related neurodegenerative conditions 94, 95, 96.
