# Supplementary material for: Circadian Disruptions in the Myshkin Mouse Model of Mania Are Independent of Deficits in Suprachiasmatic Molecular Clock Function
Source: Biol Psychiatry. 2018 Dec 1;84(11):827–37. doi: 10.1016/j.biopsych.2017.04.018 (PMC6218650; doi:10.1016/j.biopsych.2017.04.018)
Supplement: Supplemental Material [file mmc1.pdf]

Circadian Disruptions in the *Myshkin* Mouse Model of Mania Are Independent of Deficits in Suprachiasmatic Molecular Clock Function

Supplemental Information

Supplementary Materials and Methods

Animal housing and breeding: *Myk/+* animals were originally generated through an ENU mutagenesis screen and backcrossed on to the C57BL/6NCr strain for 20 generations. *Myk/+* mice at N20 C57BL/6NCr were previously reported to be free of stress-induced seizure activity during electrocorticography (1). From this same line, a *Myk/+* breeding colony was established at the University of Manchester and maintained on a C57BL/6NCrl (Charles River, Margate, UK) background. To generate mice in which the dynamic activities of the molecular clock can be monitored in tissues *ex vivo*, *Myk/+* mice were crossed with mice bearing a knock-in PER2-luciferase construct (PER2::LUC mice; (2)). +/+ x PER2::LUC (+/+PER2) and *Myk* x PER2::LUC (*Myk*/+PER2) animals were generated through crosses of heterozygous male *Myk/+* and female PER2::LUC animals. All behavioral and *in vitro* studies were performed on F1 generation animals. PER2::LUC mice have a C57BL6/J background with a fusion luciferase protein attached to the 3' end of the mammalian PER2 protein, allowing real-time bioluminescent monitoring of clock gene expression.

All animals (aged 2-6 months) were group-housed in a 12h:12h LD cycle at constant temperature (~18°C) and humidity (~40%) with food (standard lab chow) and water provided *ad libitum*. All animal protocols were in accordance with guidelines of the UK Animals (Scientific Procedures) Act 1986.

Wheel-running behavioral characterization: Adult male and female +/+ (n=37) and *Myk/+* (n=38) mice were individually housed in polycarbonate cages (410 x 247 x 124mm) equipped with 160mm diameter lined stainless steel running wheels with food and water

provided *ad libitum.* Animals were maintained in light-tight cabinets and exposed to a 12h:12h LD cycle (~56 μW/cm^2 from a broad spectrum fluorescent light source) for a minimum of 14 days before release into DD for a minimum of 14 days to assess free-running rhythms. The wheel-running rhythms of a separate cohort of female mice (+/+ n=8, *Myk*/+ n=7) were assessed as described above in LD, DD, and an 8h advanced LD (see below) as well as in constant light (LL; for up to 15 days; light intensity 56 μW/cm^2).

Behavioral assessment without running-wheels: To monitor behavior in the absence of wheel-running activity, mice (+/+ n=7, *Myk/*+ n=9) were singly-housed in cages measuring 425 x 265 x 150 mm that were equipped with a running wheel that was permanently disabled (unable to rotate) for the duration of the experiment (TSE Systems, Bad Homburg, Germany). Cages were fitted with an infrared activity monitoring system (Inframot, TSE Systems) to record locomotor activity in the homecage. Activity was recorded every 10min using PhenoMaster software (TSE Systems).

Mice were maintained under a 12h light:12h dark cycle (LD; light intensity 56μW/cm^2) for a minimum of 15 days before transfer to constant darkness (DD) for the remainder of the experiment. After an initial acclimatization period of ~5 days, behavioral activity was monitored for the last 10 days of LD and the first 10 days of DD, during which we assessed the period of activity and the duration of the active phase (alpha) under both LD and DD. Alpha was assessed from actograms using eyefit regression lines through the daily onsets and offsets of activity. Period was assessed using Clocklab software (Actimetrics, Evanston, IL, USA).

Phase shifting protocol: Animals were exposed to either an Aschoff type I (+/+ n=18; *Myk*/+ n=15) or a type II light phase shifting protocol (+/+ n=11; *Myk*/+ n=11) (3). Under type I conditions, animals were allowed to free-run in DD for 14 days prior to receiving a light pulse. On the day of the pulse, CT12 for each animal was predicted by fitting a regression line to the wheel-running actogram. At the predicted circadian time (CT; CT14, CT20, CT23),

animals were carefully transferred to a light-tight cabinet in an adjacent room with the lights on (~56 μW/cm^2) for one hour before return to home cages. For type II pulses, animals were released into DD for 24h and the cabinet light turned on for one hour (~56 μW/cm^2) at the appropriate CT, calculated relative to ZT0 under 12h:12h conditions. For this protocol, animals were not moved to another cabinet for light pulsing.

Re-entrainment protocol: To assess the rate of re-entrainment to a new light-dark cycle and hence the animal's response to 'jet-lag', male and female (+/+ n=10, *Myk*/+ n=10) animals were individually housed in a 12h:12h LD cycle for 14 days and then the LD cycle was advanced 8 hours, with lights-on now occurring some 8h earlier relative to the previous LD cycle. Animals were maintained on this advanced 12h:12h LD cycle for a further 14 days before the LD cycle was delayed by 8 hours. To delay the LD cycle, lights were kept on for 8 hours into the dark phase and the new 12h:12h LD cycle was maintained for a further 14 days. For the transient phase advance protocol (+/+ n=7, *Myk*/+ n=5), the LD cycle was advanced by 7h as described above, but this new lighting schedule was maintained for only 48h and then the animals were released into DD.

Feeding, drinking and metabolic activity assessment: Adult male +/+ (n=10) and *Myk*/+ (n=12) animals were individually housed in sealed Comprehensive Lab Animal Monitoring System (CLAMS, Columbus Instruments, Ohio, USA) Perspex metabolic cages for 7 days and maintained under a 12h:12h LD cycle at constant temperature (~18°C) and humidity (~40%). Due to local ethical restrictions, metabolic activity could not be assessed under DD conditions. Metabolic cages were connected through an open-circuit gas flow system provided with a known concentration of oxygen and carbon dioxide to allow constant, indirect calorimetric assessment. Standard lab chow was provided at an open access food hopper with an infrared beam across the opening. Feeding behavior was recorded as number of beam breaks. Water was provided *ad libitum* through a plastic water bottle in the roof of the cage with a metallic sipper. When drinking, a small current passed through the mouse and

the conductive steel-based floor and recorded on a personal computer. All activity was recorded in 10-minute bins for analysis.

Masking protocol: To assess the effects of “negative” masking on behavior, after 14 days under a 12h:12h LD cycle animals were given a 1-hour light-pulse (~56 μW/cm^2) without physical disturbance at either ZT14 or ZT20 (+/+ n=18; *Myk*/+ n=23). In addition, a cohort of +/+ and *Myk/*+ animals were exposed to an 8-hour light pulse (~56 μW/cm^2) in which lights were turned on between ZT16–ZT24.

Open-field assessment: All animals (+/+ n=8; *Myk*/+ n=7) were group housed and maintained under a 12h:12h LD cycle. Assessments were performed in a 300 x 300 x 240 mm polyethylene arena (Figure S2) and data acquired through a video camera mounted above the recording area. Animals were habituated to the arena as a group then individually over a period of 5 days prior to testing. During data collection, the experimental room was illuminated in dim light (~1.5 μW/cm^2) and each animal placed in the arena between ZT15-18 and left for 15 minutes with minimal disturbance for video acquisition (24 frames s^{-1}). Videos were analysed using EthoVision XT (Noldus, Netherlands).

Optic Nerve Crush (ONC) protocol: Mice were anaesthetised with a mixture of 13 mg/kg of Rompum™ (Bayer Inc., Mississauga, Ont., Canada) and 87 mg/kg of Ketalar® (ERFA Canada 2012 Inc., Montreal, QC, Canada). Under a dissecting microscope, a small incision was made with spring scissors in the conjunctiva, beginning inferior to the globe and around the eye temporally. To expose the posterior aspect of the globe, allowing visualization of the optic nerve, the edge of the conjunctiva next to the globe was retracted with micro-forceps, rotating the globe nasally. Using Dumont cross-action forceps, the exposed optic nerve was clamped approximately 1–3 mm from the globe for 10 s, after which the optic nerve was released and the forceps removed, allowing the eye to rotate back into place. At the end of the procedure, a drop of 0.5% proparacaine hydrochloride ophthalmic solution was administered for post-operative pain control, and a small amount of surgical lubricant (KY

jelly) was applied to the eye to protect it from drying. The mouse was placed on a warming pad and monitored until it fully recovered from anaesthesia. These experimental procedures were approved by the University of Toronto Animal Care Committee, in accordance with established guidelines of the Canadian Council on Animal Care. The effects of ONC/sham procedure on circadian rhythms were tested on mice free-running in constant dark with food and water available *ad libitum* at a room temperature at 20 ± 2°C. Animals were individually housed in polycarbonate cages equipped with 17 cm diameter running-wheels. Rotations of the wheel were continuously recorded using VitalView (Starr Life Sciences Co., Oakmont, PA., USA).

Bioluminescence imaging: Male and female *Myk*/+ x PER2::LUC (n=14) and +/+ x PER2::LUC (n=14) mice were individually-housed in wheel-running cages for 14 days under 12h:12h LD conditions and then released into DD for 14 days to monitor free-running rhythms. After 15 days under DD, animals were culled in darkness at CT8 and brains removed and immediately placed in ice-cold Hank's Buffered Saline Solution ($NaHCO_3$-supplemented HBSS, Sigma, UK) supplemented with $10mgml^{-1}$ penicillin-streptomycin (Gibco Invitrogen, Paisley, UK) and 0.01M HEPES (Sigma, UK). Brains were blocked and mid-coronal hypothalamic slices (250µm) cut using a vibratome (Campden Instruments, Loughborough, UK), and bilateral SCN explants extruded. SCN explants were transferred on to permeable 30mm, 0.4µM PTFE inserts (Millipore, Watford, UK) inside 35mm culture dishes (Corning, Tewksbury, USA) that contained 1ml luciferin-supplemented, sterile neuronal culture media; Dulbecco's Modified Eagle Medium (D-2909 Sigma, Gillingham, UK), 3.5g/l D-glucose (Sigma), 0.035% $NaHCO_3$ (Sigma), 0.1M HEPES (Sigma), B27 serum-free media (Gibco Invitrogen, UK), 0.1mM beetle luciferin potassium salt (Promega, Southampton, UK). Dishes were lined with autoclaved vacuum grease (Dow Corning Ltd, Coventry, UK) and sealed with glass UV-treated borosilicate glass, 0.13mm coverslips (VWR, Lutterworth, UK). Dishes were then transferred into light-tight incubator units (Galaxy-R+RS Biotech) maintained at 37°C and 5**%** CO_2 and left undisturbed for a minimum of 7

days. Bioluminescence was recorded as total photon counts using Photon-Multiplier Tubes (H8259/R7518P, Hamamatsu, UK) and integrated every 299s for 3 minutes. For single-cell imaging, an identical protocol was followed except slices were cut on an automated vibroslicer (7000smz, Campden Instruments, Loughborough, UK) and explants were placed in cover-slip based 35mm dishes (WPI, Hitchin, UK) with 1.1ml recording media. Culture dishes were maintained inside an LV-200 microscope (Olympus, Southend, UK) maintained at 37°C, with images taken at 30-minute exposure time using HoKaWo acquisition software (Hamamatsu, Welwyn Garden City, UK).

Whole-cell patch clamp electrophysiology: Hypothalamic coronal brain slices (250µm thick) containing the intermediate level (on the rostrocaudal axis) of the SCN were prepared from male and female +/+ (n=17) and *Myk*/+ (n=18) animals (aged 2-6 months) housed under 12h:12h LD conditions. To minimise phase-shifting associated with slice preparation, animals were culled between ZT 1-4 or ZT 10-11 (4). Whole-brains were blocked and placed in ice-cold, low Na^+, low Ca^{2+}, high sucrose, high Mg^{2+} artificial cerebrospinal fluid (aCSF); NaCl 95mM; KCl 1.8mM; KH_2PO_4 1.2mM; $CaCl_2$ 0.5mM; $MgSO_4$ 7mM; $NaHCO_3$ 26mM; Glucose 15mM; Sucrose 50mM; Phenol Red 0.005mg L^{-1}; pH 7.4; 300-310 mOsmol kg^{-1} pre-bubbled with 95% O_2; 5% CO_2. 250µm thick slices were cut on an automated vibroslicer (7000smz, Campden Instruments). Slices were then transferred to the recording chamber mounted on the stage of an upright Olympus microscope (BX51WI, Olympus, Essex, UK) and continuously perfused (~3ml/min) with room temperature (20-23°C) recording aCSF; NaCl 127mM; KCl 1.8mM; KH_2PO_4; 1.2mM; $NaHCO_3$; 26mM; $CaCl_2$ 2.4mM; $MgSO_4$ 1.3mM; Glucose 15mM; Phenol Red 0.005mg ml^{-1}; pH 7.4; 300-310 mOsmol kg^{-1}) bubbled with 95% O_2; 5% CO_2.

Whole-cell current-clamp recordings were made using a npi BA-01X bridge amplifier (npi electronics, Tann, Germany). Recording electrodes were fashioned from borosilicate glass capillaries pulled on a two-stage pipette puller (PC-10, Narishige, Tokyo, Japan). Pipettes (7-10MΩ) were half-filled with 0.22µm filtered intracellular solution; K-gluconate 130mM; KCl

10mM; $MgCl_2$ 2mM; K_2-ATP 2mM; Na-GTP 0.5mM; HEPES 20mM; EGTA 0.5mM; pH 7.28 with KOH; Osmolarity 295-300mOsmol kg^{-1}; stored on ice to prevent ATP and GTP degradation. Cells were targeted using infrared video-enhanced differential interface contrast microscopy using a 40x water-immersion lens. Signals were sampled at 30 KHz and stored on a personal computer running Spike2 software for analysis (Cambridge Electronic Design, Cambridge, UK).

Calcium imaging: Coronal hypothalamic brain slices (200µm thickness) were prepared as described for patch-clamp recordings for assessment during the day (ZT4-10: +/+ n=3; *Myk*/+ n=3) or the night (ZT14-18: +/+ n=5; *Myk*/+ n=4). Slices were immediately transferred into recording aCSF bubbled with 95% O_2; 5% CO_2 and incubated in leak-resistant Fura-2(AM) (TefLabs, Austin, USA) dissolved in 50µl Pluronic F-127; 20% solution in DMSO (Molecular Probes, Life Technologies, USA) at 37°C for 15 minutes, then at room temperature (20-23°C) for one hour. Once loaded, slices were perfused continuously (2.5ml min^{-1}) with recording aCSF for 1 hour for de-esterification prior to measurement of intracellular Ca^{2+}. Imaging of intracellular calcium levels in single neurons and their responses to pharmacological stimulation was performed on an upright Olympus BX51 WI microscope mounted to a vibration-free air table (63-500 series, TMC, MA, USA). Image capture was performed using a water-immersion UV objective (UMplanFL N 20x/0.5, Olympus, Japan) and dual-excitation from two opto-LEDs (Cairn Research, Kent, UK) at 365nm and 385nm every 2 seconds. Excitation emissions were filtered at 510nm and captured by a cooled, high-sensitivity QImaging Rolera EM C2 CCD camera (QImaging, Surrey, B.C., Canada) connected to a PC running Optofluor Version 7.7.5.0 (Cairn Research) for offline analysis.

AMPA (Tocris; 5µM, 10µM and 20µM) was bath applied via a gravity-driven perfusion system for 60s and 90s, respectively. Responses to drug application were defined as a ratio change of >2 S.D. of 10 consecutive background-subtracted time points from manually-determined somatic regions of interest of individual neurons.

Immunohistochemistry: Whole-brain tissue was fixed in PFA and frozen in dry ice. 35μm slices were cut using a sledge microtome and placed in 0.1M PBS solution. Slices were blocked in 5% donkey serum (Sigma) and 0.1% Triton X-100 then incubated for 48h with primary antibodies (Rabbit polyclonal anti-VIP 1:1000, Enzo Life Sciences, NY, USA; Rabbit polyclonal anti-AVP 1:5000, EMD Millipore, CA, USA) in 0.5% donkey serum, 0.05% Triton X-100. Slices were washed and incubated in secondary antibody (Cy-3 conjugated AffiniPure Donkey anti-rabbit IgG 1:800, Jackson, PA, USA) without donkey serum for 24h then mounted with Vecta-shield media (Vector Laboratories, CA, USA).

Data analysis and statistics: Data were analysed and statistics applied in Prism6 (Graphpad, La Jolla, CA, USA), Origin Pro 9.0 (Originlab, Northampton, MA, USA) and SPSS version 16 (SPSS Inc, Chicago, USA). All data are presented as mean plus standard error. Unless stated otherwise, genotype comparisons were made using two-tailed Student's t-test or by two-way ANOVA with Sidak post-hoc comparisons. For within genotype comparisons, a one-way ANOVA was applied with Sidak corrections unless stated. Non-parametric equivalents and corrections for unequal variances were utilised where appropriate and are detailed in figure captions.

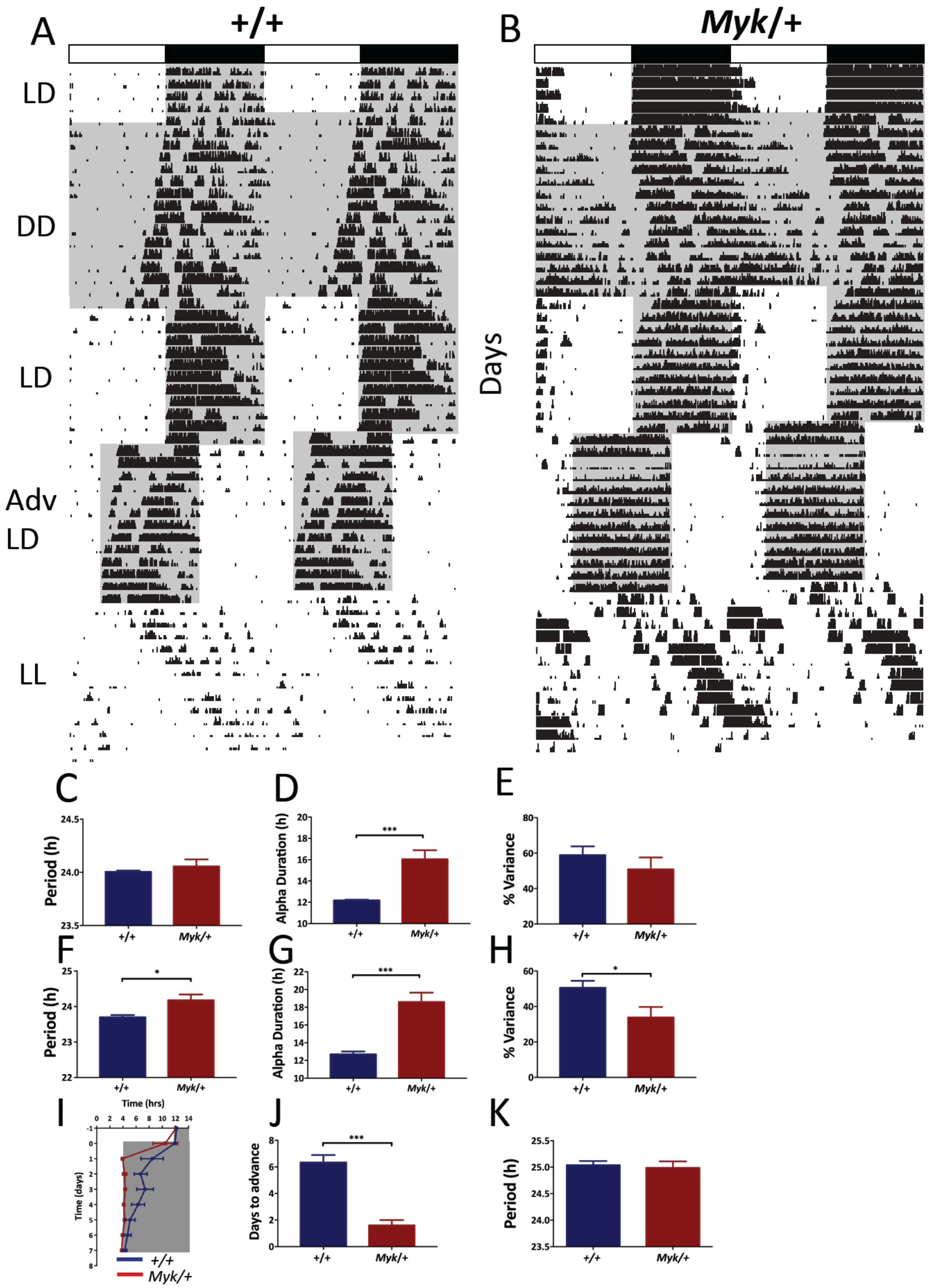

Figure S1: Female *Myk*/+ mice exhibit altered daily and circadian rhythms in wheel-running activity.

A, B. Representative double-plotted wheel-running actograms of female +/+ and *Myk*/+ mice respectively under light-dark (LD), constant dark (DD), 8h advanced LD cycle (Adv LD), and constant light (LL). Under the initial LD, no genotype differences were seen in **C**. period,

whereas compared to +/+ animals, **D**. alpha duration increased (+/+ 12.18 ± 0.05h vs *Myk*/+ 16.05 ± 0.85h, p=0.0005) and **E**. % Variance (rhythm strength) was slightly decreased (+/+ 58.9 ± 4.9% vs *Myk*/+ 50.9 ± 6.7%, p>0.05) in the *Myk*/+ animals. In DD, *Myk*/+ mice exhibited significantly longer **F**. circadian period (+/+ 23.7 ± 0.06h vs Myk/+ 24.18 ± 0.16h, p=0.12) and **G**. alpha (+/+ 12.7 ± 0.32h vs 18.6 ± 1.0h, p<0.0001) and **H**. reduced rhythm strength (+/+ 50.6 ± 3.9% vs 33.9 ± 5.9%, p=0.032). In response to an 8h advance in the LD cycle, *Myk*/+ animals **I,J**. re-synchronized the onset of wheel-running activity in significantly fewer days than +/+ mice. In LL, *Myk*/+ animals **K**. increased wheel-running, whereas +/+ mice reduced this activity. The % change in wheel-running/h (LD to LL) was significantly different between the genotypes (Mann-Whitney U test, p=0.006). *p<0.05, ***p<0.001.

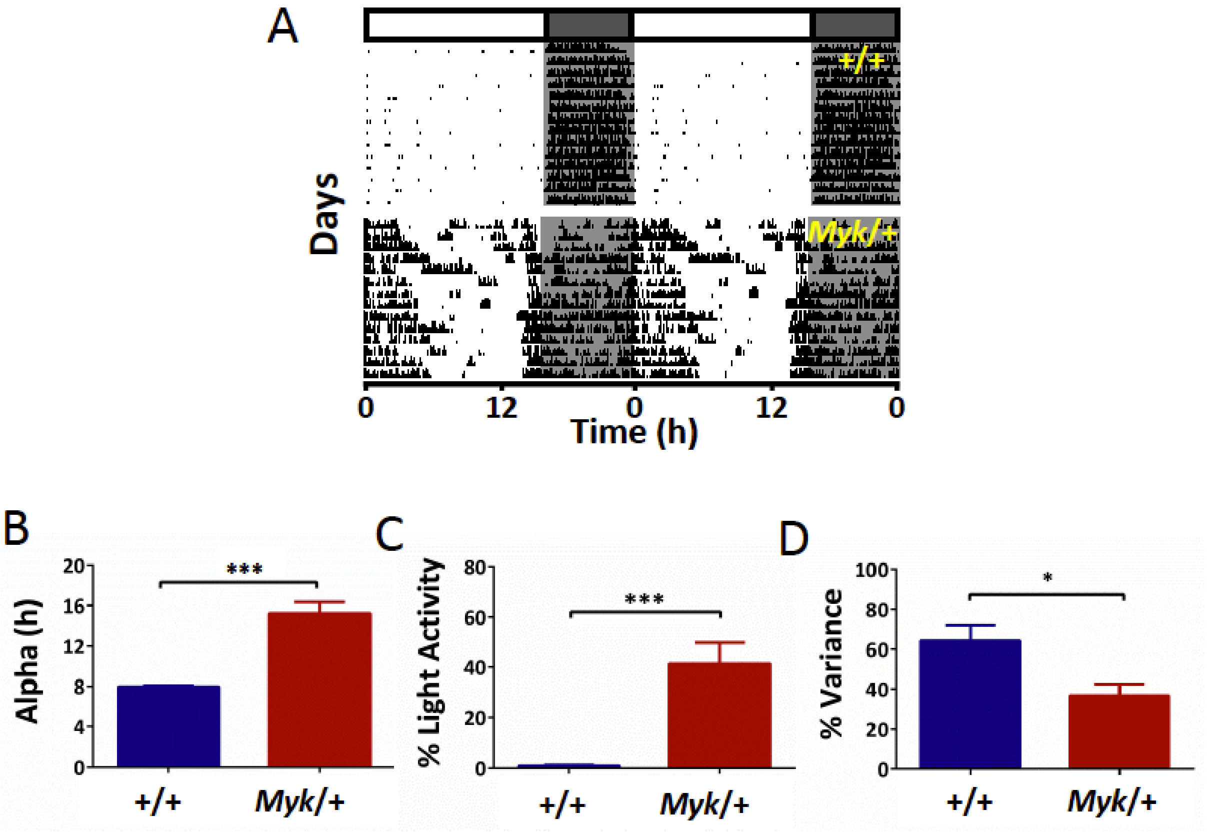

Figure S2: Poor consolidation of *Myk*/+ behavioral rhythms to the dark phase during extended daylength (16h light: 8h dark).

A: Example double-plotted actograms of +/+ (n=8) and *Myk*/+ (n=7) animals maintained under long day 16h:8h LD conditions. Gray shaded areas of actograms delineate lights-off (dark). **B**: Mean alpha duration is significantly longer in *Myk*/+ animals. (+/+: 8.0 ± 0.1h; *Myk*/+: 15.2 ±1.1h, p<0.0001). **C**: *Myk*/+ mice show a higher percentage of total wheel-running activity within lights-on phase (+/+: 0.9 ± 0.3% *Myk*/+: 41.3 ± 8.5%, p=0.0002). **D**: The amplitude of the activity rhythm measured by χ2 periodogram is damped by the *Myshkin* mutation under 16h:8h lighting conditions (+/+: 64.3 ± 7.8%; *Myk*/+: 36.7 ± 5.8%, p=0.02). Data in B-D plotted as mean ± SEM. *p<0.05, ***p<0.001.

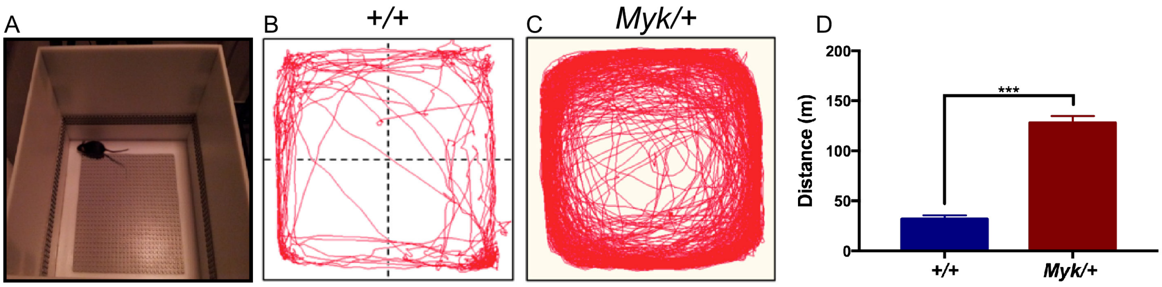

Figure S3: The *Myk/+* mice exhibit hyperlocomotor activity in an illuminated open-field when tested during subjective night (ZT15-18).

A: Experimental set-up from open-field paradigm. Image taken from main data acquisition camera. **B-C**: Example traces of +/+ (n=8) and *Myk*/+ (n=7) locomotor activity over 15 minutes in an illuminated open-field. **D**: Increased locomotion in *Myk*/+ animals in open-field test during subjective night (+/+: 32.0 ± 3.6 m, *Myk*/+: 127.9 ± 6.9 m, p<0.0001). Data in D plotted as mean ± SEM. ***p<0.001.

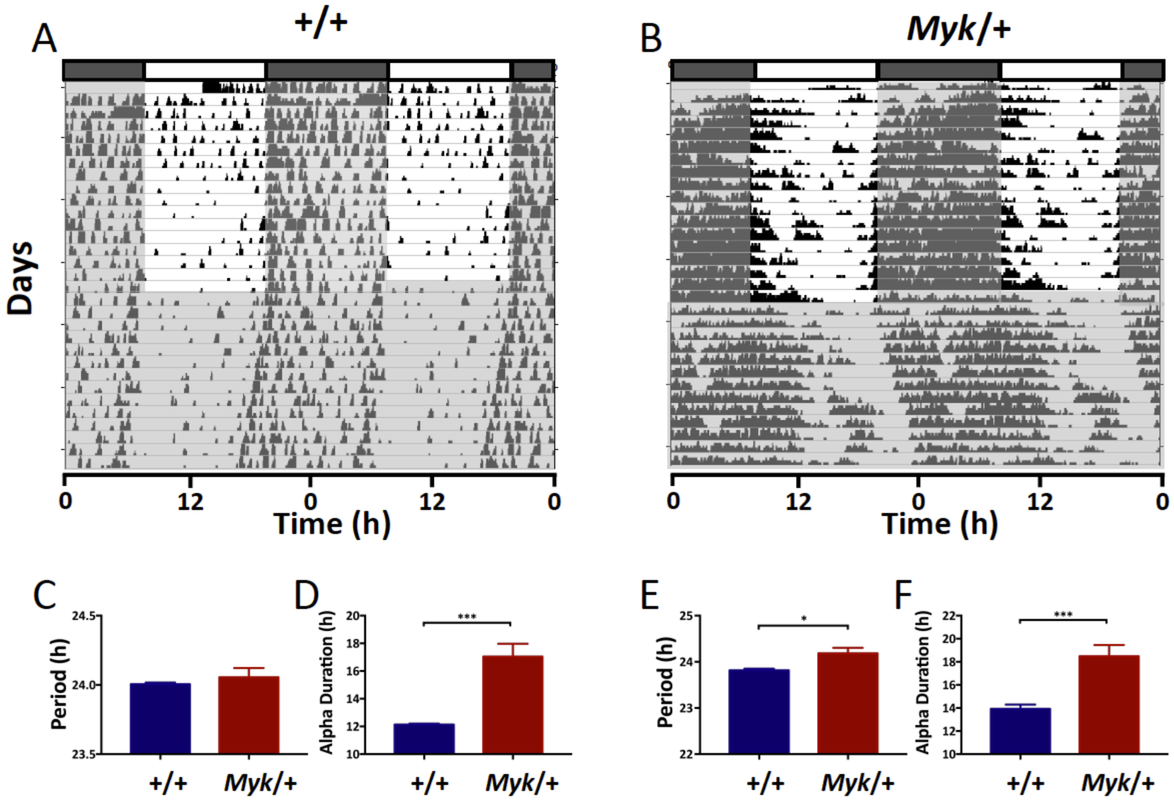

Figure S4: Effects of the *Myshkin* mutation on daily and circadian rhythms of locomotor activity are sustained in the absence of a running-wheel.

A,B. Representative double-plotted actograms of +/+ and *Myk*/+ mice respectively recorded under light-dark (LD) and constant dark (DD) conditions with a passive infrared detector. Under LD, no genotype differences were seen in **C**. period, whereas **D**. the duration of the active phase (alpha) was significantly prolonged in *Myk*/+ compared with +/+ animals (17.05 ± 0.9h vs 12.1 ± 0.07h, p=0.0007). When free-running in DD, **E**. circadian period (24.18 ± 0.12h vs 23.82 ± 0.04h, p=0.015) and alpha (18.49 ± 0.9h vs 13.9 ± 0.4h, p=0.0008) were significantly longer in *Myk*/+ compared with +/+ mice. *p<0.05, ***p<0.001.

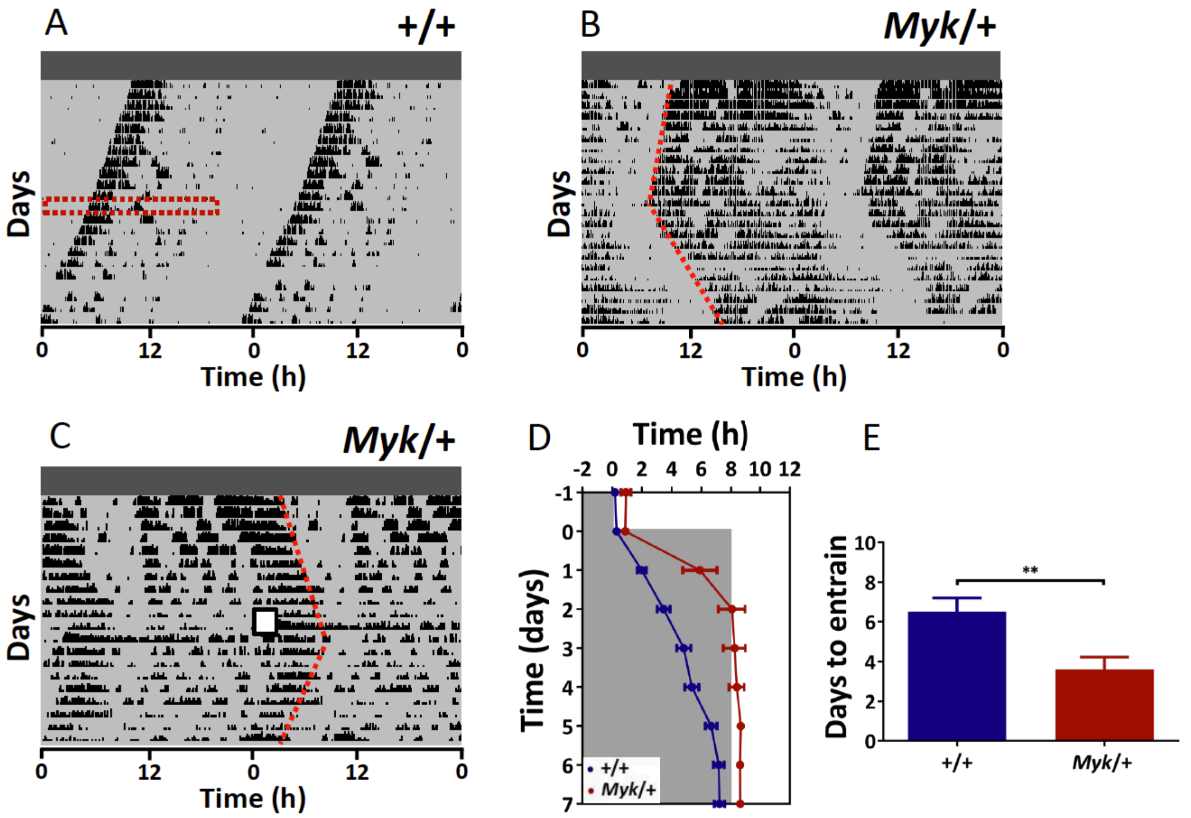

Figure S5: *Myk*/+ animals exhibit unstable free-running circadian rhythms in constant dark (DD) as well as rapid re-entrainment to an 8h delay in the light-dark cycle.

Abrupt changes in free-running period arising spontaneously or elicited by arousal respectively are illustrated in the double-plotted actograms (**A-C**). **A**: Wheel-running rhythm of +/+ animal maintained in DD during same experiment as (**B**) a *Myk*/+ animal that exhibits a spontaneous change in free-running period highlighting innate instability of rhythms. Red rectangle in (**A**) indicates day on which a spontaneous change in circadian period occurred in the *Myk*/+ animal, but not the +/+ mouse. **C**: *Myk*/+ animal showing a shortening of circadian period after cage base was refreshed with new bedding (white square). **D**: *Myk*/+ mice show rapid re-entrainment to an 8h delay in the timing of the LD cycle. **E**: Mean phase shift of +/+ and *Myk*/+ animals to 8h delay of LD cycle. Gray shading in A-D depicts lights-off (dark). Data in D and E are plotted as mean ± SEM. **p<0.01.

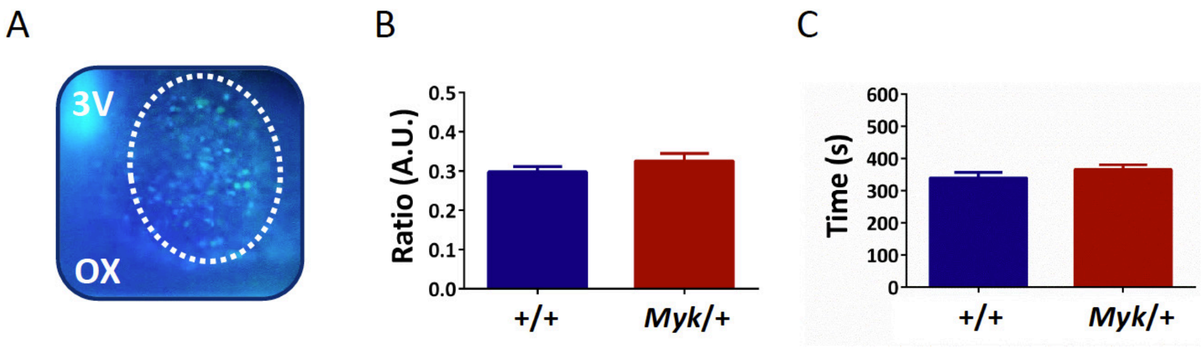

Figure S6: Intracellular calcium responses to daytime application of AMPA.

A: Example false-colour image of unilateral SCN loaded with Fura-2 AM. **B**: During the subjective day (ZT4-10), AMPA (20μM) evoked change in intracellular Ca^{2+} of individual SCN neurons did not differ between the genotypes (+/+ n=142, 0.29 ± 0.01 A.U; *Myk*/+ n=109, 0.32 ± 0.02 A.U.; p=0.22). **C**: During the subjective day (ZT4-10), the washout duration of AMPA responses were similar in both genotypes (+/+*:* 340 ± 17s, *Myk*/+: 365 ± 15s, p=0.27). Data in B and C are plotted as mean ± SEM.

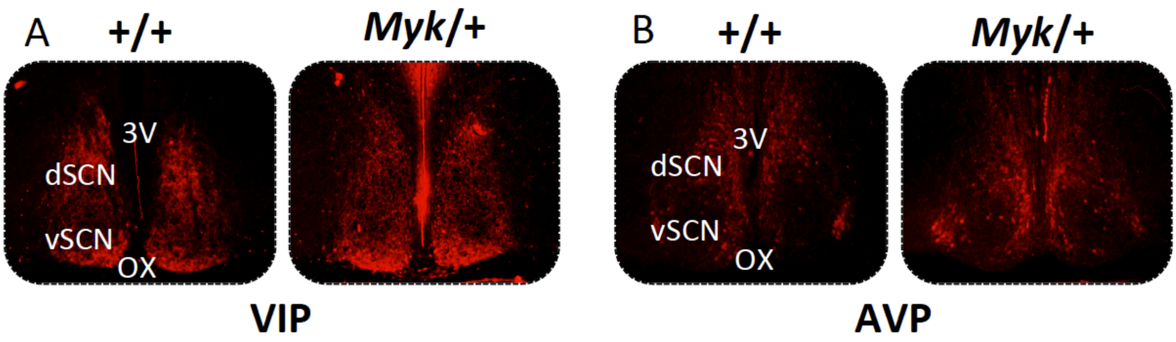

Figure S7: Anatomically distinct distribution of two key neuropeptides in the SCN does not vary between *Myk*/+ and +/+ mice.

A: Mid-coronal SCN slices from +/+ and *Myk*/+ animals immunohistochemically stained with anti-vasoactive intestinal polypeptide (VIP) primary antibody. Cells immunoreactive for VIP are localized to the ventral SCN (vSCN) in both genotypes, while VIP immunoreactive processes are present in the dorsal SCN (dSCN). **B**: Mid-coronal SCN slices stained with anti-arginine vasopressin (AVP) primary antibody. AVP immunoreactive cells are present in the dSCN as well as in the lateral aspect of the vSCN. No overt genotype-related differences in the patterns of immunostaining for these two neuropeptides were observed. Note the SCN are bilateral, with one lobe on either side of the floor of the third ventricle (3v), and are positioned above the optic chiasm (OX).

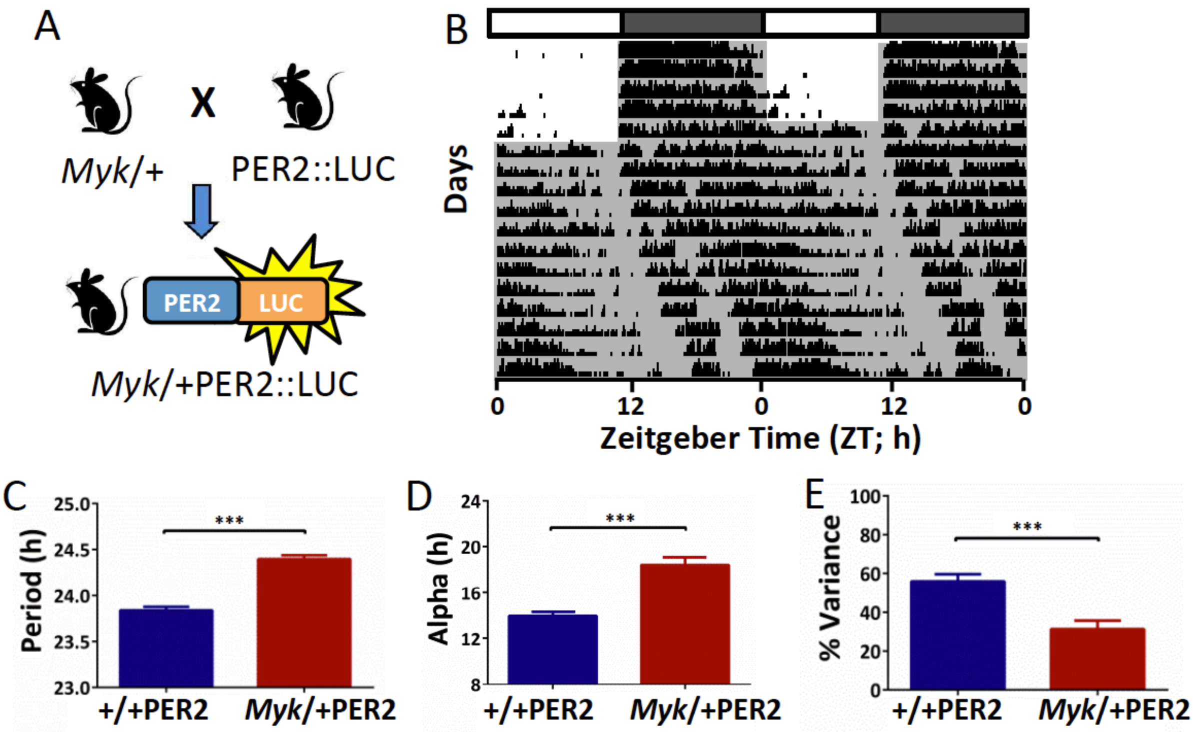

Figure S8: Circadian rhythms in wheel-running are altered in the *Myk*/+PER2 mouse and strongly resemble observations made in *Myk*/+ animals.

A: +/+ and *Myk*/+ animals were crossed on to PER2::LUC bioluminescent reporter background. **B**: Example double-plotted actogram of F1 progeny *Myk*/+PER2 mouse released into free-running conditions. Gray shaded area of actogram delineates lights-off (dark). Period (**C**), activity duration (**D**) and rhythm strength (%Variance) (**E**) of DD free-running rhythms in +/+PER2 and *Myk*/+PER2 mice. **C**: (Period: +/+PER2: 23.84 ± 0.04h; *Myk*/+PER2: 24.40 ± 0.04h, p<0.0001). **D**: (Alpha: +/+PER2: 13.99 ± 0.35h; *Myk*/+PER2: 18.43 ± 0.66h, p<0.0001). **E**: Rhythm strength (+/+PER2: 55.8 ± 3.7%Var; *Myk*/+PER2: 31.26 ± 4.4%Var, p=0.0005). The characteristics of wheel-running rhythms of *Myk*/+PER2 animals are very similar to those of *Myk*/+ animals. Data in C-E plotted as mean ± SEM. ***p<0.001.

Table S1: Parameters of comparable electrical states of SCN neurons detected in *Myk*/+ and +/+ animals.

The *Myshkin* mutation does not affect the parameters of SCN neuronal states

Cell State	Number of Cells	RMP (mV)	SFR (Hz)
Regular Firing	+/+ (n=37)<br>*Myk*/+ (n=38)	-42.4 ± 0.9mV<br>-43.9 ± 0.6mV	3.1 ± 0.2Hz<br>3.0 ± 0.1Hz
Irregular Firing	+/+ (n=42)<br>*Myk*/+ (n=42)	-40.8 ± 0.6mV<br>-41.3 ± 0.5mV	1.4 ± 0.1Hz<br>1.3 ± 0.1Hz
Depolarised	+/+ (n=22)<br>*Myk*/+ (n=24)	-33.8 ± 0.7mV<br>-33.4 ± 0.5mV	NA<br>NA
Hyperpolarised	+/+ (n=34)<br>*Myk*/+ (n=32)	-51.0 ± 0.7mV<br>-51.9 ± 1.0mV	NA<br>NA

Supplemental References

1. Kirshenbaum GS, Clapcote SJ, Duffy S, Burgess CR, Petersen J, Jarowecek KJ, Yucel YH, Cortez MA, Snead III CO, Vilsen B, Peever JH, Ralph MR, Roder JR (2011) Mania-like behaviour induced by genetic dysfunction of the neuron-specific Na^{+},K^{+}ATPase α3 sodium pump. *Proc. Natl. Acad. Sci. USA* 108: 18144-18149.

2. Yoo S-H, Yamazaki S, Lowrey PL, Shimomura K, Ko CH, Siepka SM, Hong H-K, Oh WJ, Yoo OJ, Menaker M, Takahashi JS (2004) PERIOD2::LUCIFERASE real-time reporting of circadian dynamics reveals persistent circadian oscillations in mouse peripheral tissues. *Proc. Natl. Acad. Sci. USA* 101:5339–5346.

3. Jud C, Schmutz I, Hampp G, Oster H, Albrecht U (2005) A guideline for analyzing circadian wheel-running behaviour in rodents under different lighting conditions. *Biol. Proced. Online* 7: 101-116.

4. Gillette MU, Medanic M, McArthur AJ, Liu C, Ding JM, Faiman LE, Weber ET, Tcheng TK, Gallman EA (1995): Intrinsic neuronal rhythms in the suprachiasmatic nuclei and their adjustment. *Ciba Found Symp*. 183: 134–44.
